# Supplementary material for: Comprehensive and Accurate Molecular Profiling of Breast Cancer through mRNA Expression of ESR1, PGR, ERBB2, MKI67, and a Novel Proliferation Signature
Source: Diagnostics (Basel). 2024 Jan 23;14(3):241. doi: 10.3390/diagnostics14030241 (PMC10855423; doi:10.3390/diagnostics14030241)
Supplement: Supplementary file 1 [file diagnostics-14-00241-s001.zip › Supplementary File S1 - Repeatability and Reproducibility Study Design.pdf]

Supplementary File S1: Repeatability and Reproducibility Study Design

1. Study Design

The aims of the study were to determine the performance attributes – precision (variability) namely repeatability (within run precision), intermediate precision (within site precision) and reproducibility (between site precision) for the APIS BC Subtyping kit.

Repeatability was estimated by generating replicate measurements of the same samples using an identical PCR layout, using the same protocol, operators, instrument, and location over a short period of time.

Within site (or Intermediate) precision was generated using replicate measurements of the same samples using an identical PCR layout under the same operating conditions. Between run, within day, and within device precision was estimated.

Reproducibility was estimated by generating replicate measurements of the same samples using an identical PCR layout, protocol, but using different operators, locations, and instruments.

Three sites were used to assess reproducibility, APIS Assay Technologies and two external sites.

The study design was based on the CLSI guidelines (EP05-A3).

2. Sample Preparation

Test samples were prepared at site 1 (APIS Assay Technologies) using RNA extracted from FFPE Breast Cancer specimens. For each target two challenging samples were prepared – 'low' positive and 'medium' positive. A negative sample for each target was also prepared.

To contrive samples RNA pools for each target were prepared and combined with a negative expression RNA pool in a series of 1:1 to 1:5 ratio's and tested by PCR to determine the correct ratio of positive and negative sample to result in the desired ΔCt for the target. Low and mid positive expression samples are defined as generating ΔCt 1.5-2.0 ΔCt and 2.5-3.0 ΔCt above the target cut-off respectively. Details of the samples contrived can be found in Table 1.

Table 1. Samples prepared for R&R studies.

Sample	Aliquot Volume	Concentration	Target expression
S1	100µL	2.5ng/µL	Negative ESR1 and PGR, medium positive ERBB2
S2	100µL	2.5ng/µL	Medium positive ESR1 and PGR, negative (low) MKI67
S3	100µL	2.5ng/µL	Negative ERBB2
S4	100µL	2.5ng/µL	Low positive ESR1 and PGR

S5	100µL	2.5ng/µL	Low positive ERBB2, medium positive MKI67
S6	100µL	2.5ng/µL	Low positive MKI67

3. Study Workflow

For each site two kit lots were prepared and stored at -20°C in a temperature controlled and monitored freezer. A total of three kit lots were used in this study.

For site 1 (Apis), 20 runs performed across 10 non-consecutive days. For sites 2 and 3, 10 runs were performed across five non-consecutive days. At all sites, two runs were performed on each day, using two different plate templates (containing different samples) and two operators. One QS5 Dx instrument was used at each external site, and two instruments were used at the central site (Apis) (Figure 1). All testing was perfromed following APIS BC Subtyping Kit Instruction for Use.

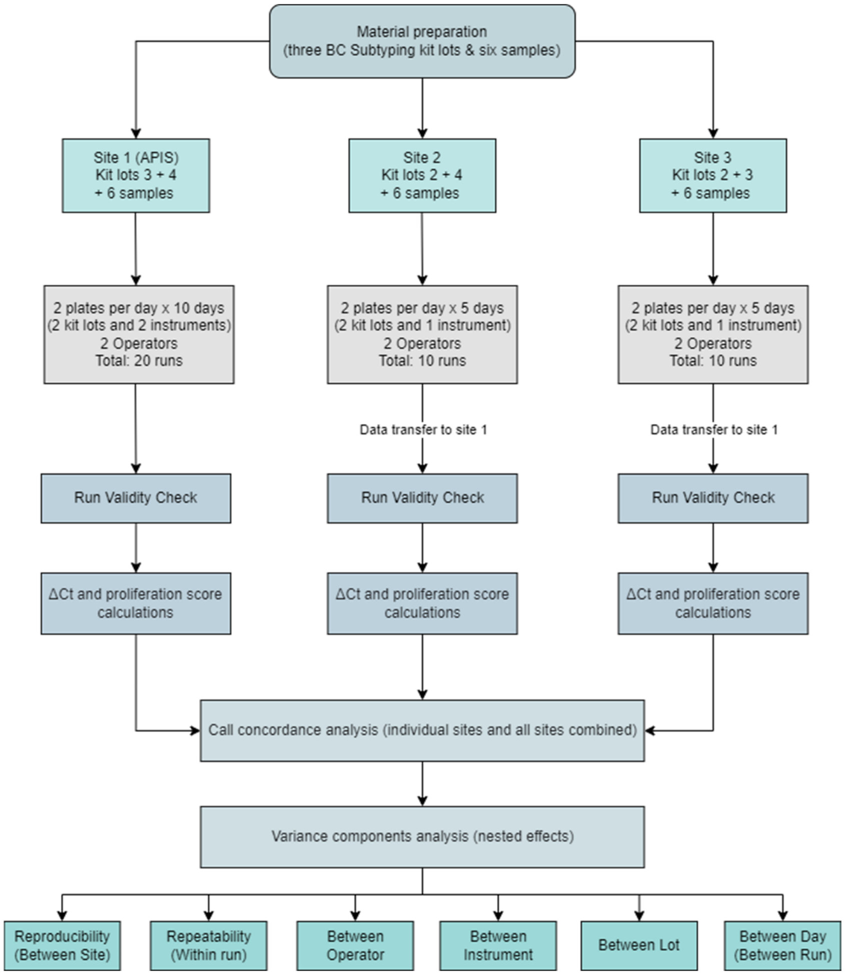

Figure 1. Process diagram for repeatability and reproducibility study
