# Supplementary material for: Comprehensive and Accurate Molecular Profiling of Breast Cancer through mRNA Expression of ESR1, PGR, ERBB2, MKI67, and a Novel Proliferation Signature
Source: Diagnostics (Basel). 2024 Jan 23;14(3):241. doi: 10.3390/diagnostics14030241 (PMC10855423; doi:10.3390/diagnostics14030241)
Supplement: Supplementary file 1 [file diagnostics-14-00241-s001.zip › Supplementary File S2 - Withdrawn Samples.pdf]

Supplementary File S2: Withdrawn Samples

The samples excluded from cohort 2, and the reasons for withdrawal from the study can be found in Table 1.

Table 1. Reasons for withdrawal of clinical specimens

Reason for withdrawal	# Samples
Adenoma	1
Mainly or entirely DCIS	75
Diagnostic data incomplete	1
Insufficient material quantity	26
No or insufficient tumour content (<20)	89
Liver metastasis	2
Lymph nodes	19
Not breast cancer	2
Not clearly invasive	9
Pleural effusion	1
Usual Ductal Hyperplasia (UDH)	2
Undergone decalcification	2
Total	229
